# Ultrastructural observations on the oncomiracidium epidermis and adult tegument of *Discocotyle sagittata*, a monogenean gill parasite of salmonids

**DOI:** 10.1007/s00436-020-07045-z

**Published:** 2021-01-12

**Authors:** Mohamed Mohamed El-Naggar, Richard C Tinsley, Jo Cable

**Affiliations:** 1 Zoology Department, Faculty of Science, Mansoura University, Mansoura, Egypt; 2 School of Biosciences, Cardiff University, Cardiff, CF10 3AX UK; 3 School of Biological Sciences, University of Bristol, Woodland Road, Bristol, BS8 1UG UK

**Keywords:** Monogenea, Tegument, Ultrastructure, Oncomiracidium, Salmonids, Aquaculture

## Abstract

During their different life stages, parasites undergo remarkable morphological, physiological, and behavioral “metamorphoses” to meet the needs of their changing habitats. This is even true for ectoparasites, such as the monogeneans, which typically have a free-swimming larval stage (oncomiracidium) that seeks out and attaches to the external surfaces of fish where they mature. Before any obvious changes occur, there are ultrastructural differences in the oncomiracidium’s outer surface that prepare it for a parasitic existence. The present findings suggest a distinct variation in timing of the switch from oncomiracidia epidermis to the syncytial structure of the adult tegument and so, to date, there are three such categories within the Monogenea: (1) Nuclei of both ciliated cells and interciliary cytoplasm are shed from the surface layer and the epidermis becomes a syncytial layer during the later stages of embryogenesis; (2) nuclei of both ciliated cells and interciliary syncytium remain distinct and the switch occurs later after the oncomiracidia hatch (as in the present study); and (3) the nuclei remain distinct in the ciliated epidermis but those of the interciliary epidermis are lost during embryonic development. Here we describe how the epidermis of the oncomiracidium of *Discocotyle sagittata* is differentiated into two regions, a ciliated cell layer and an interciliary, syncytial cytoplasm, both of which are nucleated. The interciliary syncytium extends in-between and underneath the ciliated cells and sometimes covers part of their apical surfaces, possibly the start of their shedding process. The presence of membranous whorls and pyknotic nuclei over the surface are indicative of membrane turnover suggesting that the switch in epidermis morphology is already initiated at this stage. The body tegument and associated putative sensory receptors of subadult and adult *D. sagittata* are similar to those in other monogeneans.

## Introduction

Most monogeneans are ectoparasitic while a few genera are endoparasites of fish or other vertebrates. In aquaculture, such parasites can cause significant welfare issues and economic loss (Ogawa and Timi 2015; Trujillo-González et al. 2018; EL-Naggar et al. 2019). The polyopisthocotylean *Discocotyle sagittata* (Leukart, 1842) Diesing, 1850 is an oviparous, blood-feeding, monogenean that infests the secondary gill lamellae of salmonids e.g., rainbow trout, *Oncorhynchus mykiss*Walbaum and brown trout, *Salmo trutta L*. Paling (1965) first studied the ecology of this parasite on brown trout and char in the Lake District (UK), and subsequent work included: Owen (1970), Valtonen et al. (1990), Gannicott and Tinsley (1997, 1998a, 1998b), and Rubio-Godoy and Tinsley (2002); the more recent studies sparked by disease outbreaks on fish farms. High infection of *D. sagittata* on farmed trout causes pale gills, reduced body condition, and even host mortality, but at least some resistance to the parasite is apparent (Rubio-Godoy and Tinsley 2008).

Previous ultrastructural studies on the tegument of adult monogeneans show that it consists of an outer anucleate syncytial cytoplasmic layer connected by cytoplasmic connections to nucleated subtegumental cell bodies lying among parenchymal cells beneath the superficial muscle layers (e.g., Smyth and Halton 1983; Tyler and Tyler 1997; El-Naggar et al. 1991; Ramasamy et al. 1995; Cribb et al. 2003; Hodová et al. 2010; Poddubnaya et al. 2016). In contrast, the outer body covering of the oncomiracidia is an epidermis, which is often ciliated (Whittington et al. 1999). Only a few transmission electron microscope (TEM) studies have investigated the body surface of monogenean larvae; for monopisthocotyleans, this includes *Entobdella soleae*(see Lyons 1973), *Euzetrema knoepffleri*(see Fournier 1976), *Monocotyle spiremae*, and *Neoheterocotyle rhinobatidis*(see Rohde et al. 1998), and those on polyopisthocotylean larvae are confined to a few species of the Polystomatidae (Fournier 1979; Cable and Tinsley 1992) and *Zeuxapta seriolae*(see Rohde 1998). These are probably not the most representative species of Monogenea: *E. knoepffleri* is adapted to living in the bladder of a urodele, and the polystomatids are adapted to a mesoparasitic life in amphibians and reptiles. Perhaps, not surprisingly the epidermis of *E. knoepffleri* was found to resemble that of the polystomatids. Whittington et al. (1999) recommended further studies on the oncomiracidial epidermis of more species of both monogenean subclasses, particularly those infecting fish.

Rubio-Godoy et al. (2003) reported a rapid killing of *D. sagittata* oncomiracidia, if incubated in naïve plasma from rainbow trout or brown trout and their scanning electron microscope study revealed a breached epidermis. The only other ultrastructural study conducted on this parasite was that of Cable and Tinsley (2001) who documented spermiogenesis. The present study was conducted to describe the epidermis of the oncomiracidium and tegument of subadult and adult *D. sagittata* using TEM with the aim of comparing the effects on this transition phase with other monogeneans. It was also an opportunity to assess the tegumental ultrastructure of adults that were occasionally expelled from the gills and recovered in screening water in order to record possible changes that may occur in the tegument after their detachment from the host gills. Finally, the present study provided another opportunity to clarify different types of putative sensory structures associated with the epidermis of the oncomiracidium and tegument of adult.

## Materials and methods

Rainbow trout (*Oncorhynchus mykiss*) infected with *Discocotyle sagittata* were caught at a Government Fish Hatchery in Cornaa on the Isle of Man, UK, during the summer of 1994. Fish were transported to Bristol University and maintained in aquaria according to the methods outlined by Gannicott and Tinsley (1997). Parasite eggs were collected every 24 h by draining the water from aquaria through a 125-μm sieve; the residue was resuspended in dechlorinated water and decanted into 200-ml crystallizing dishes. Using a dissecting microscope, eggs were collected into Petri dishes and incubated for 3–4 weeks at 13 ± 0.5 °C. Recently emerged oncomiracidia were fixed for TEM or used to infect naive hosts (see Gannicott and Tinsley 1998b). The silver nitrate staining technique was used to reveal boundaries of ciliated cells (Lynch 1933).

Infected fish were pithed, each gill arch was transferred quickly to a Petri dish of dechlorinated water, and parasites of different developmental stages were separated and fixed individually for TEM. Individuals of *D. sagittata* maintained on their host in aquaria were occasionally expelled from the gills and recovered in screening water. Two such adults and two subadults (with a single pair of clamps on the opisthaptor) found alive in screening water were fixed and processed for TEM. For comparison, a further two (apparently healthy) adults were removed from their host, left in dechlorinated water at 13 °C for 24 h (equivalent to the time spent off the host by the naturally expelled parasites), and then fixed for TEM. In addition, 9 larval stages (7 newly hatched oncomiracidia and 2 post-larvae retrieved from their host 24-h post-infection) were fixed together with 7 adults and 4 subadults that were processed immediately after recovery from their hosts. All specimens were fixed at 4 °C in 2.5% glutaraldehyde buffered with 0.1 M sodium cacodylate, washed overnight in the same buffer, post-fixed for 1 h in cacodylate buffered 1% osmium tetroxide and washed again in buffer, dehydrated in ethyl alcohol, and embedded in Araldite resin. Ultrathin sections were double stained with uranyl acetate and lead citrate and viewed on a JEOL 1200 EX or 1210 electron microscope operated at 80 KV.

### Ethics

Ethical considerations followed University of Bristol guidance existing at that time and studies were regulated by a UK Home Office Licence.

## Results

### Epidermis of the oncomiracidium

Hatched oncomiracidia of *Discocotyle sagittata* measure 350 (240–430) μm in length and 120 (100–170) μm in maximum width. Silver nitrate staining revealed the boundaries of 28 ciliated cells, arranged in six regions (two anterolateral, two mediolateral, and two posterior). These cells are bilaterally symmetrical and distributed as follows: five on each anterolateral region, six on each mediolateral region, and three cells in each posterior group.

**Fig. 1:**
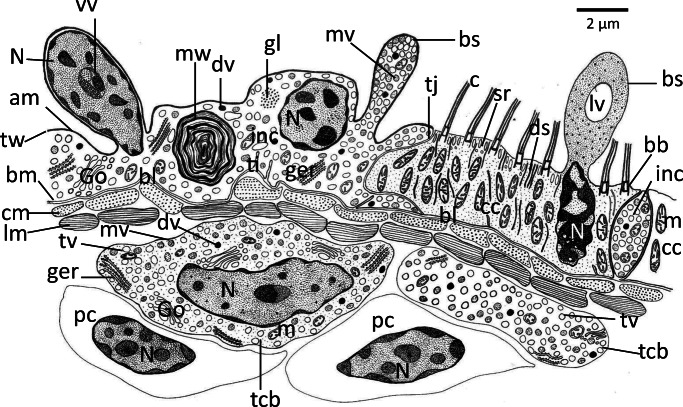
Schematic drawing showing ultrastructure of the epidermis of the oncomiracidium of *Discocotyle sagittata* in a longitudinal section. am, apical membrane; bb, basal body; bm, basal plasma membrane; bl, basal lamina; bs, bulb-like structure; c, cilium; cc, ciliated cell; cm; circular muscle fiber; ds, dense strands; dv, highly electron-dense vesicle; Go, Golgi body; gl, glycogen particles; ger, granular endoplasmic reticulum; inc, interciliary cytoplasm; lm, longitudinal muscle fiber; lv, large vacuole; m, mitochondria; mv, moderately electron-dense vesicle; mw, multilayered whorls; N, nucleus; pc, parenchymal cell; sr, striated rootlet; tcb, tegumental cell body; ti, tubular invaginations of basal plasma membrane; tj, tight junction; tv, translucent vesicle; tw, terminal web; vv, vesiculated vacuole. Scale bar = 2 μm

**Fig. 2:**
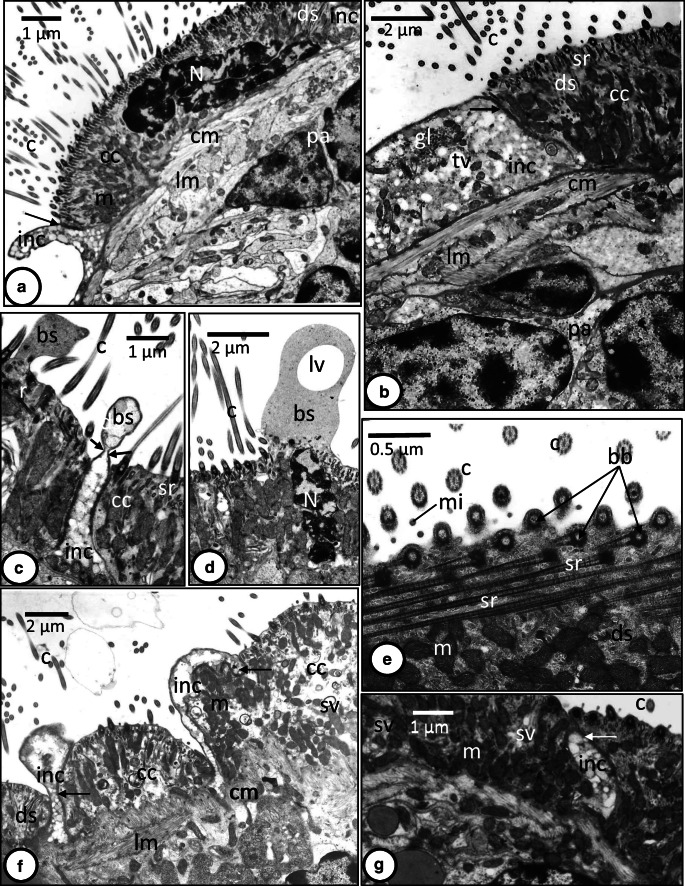
**a**‑**g** TEM micrographs of ciliated cells in the oncomiracidium of *Discocotyle sagittata*. **a** The ciliated cell (cc) joins the interciliary syncytium (inc) by tight junctions (arrow) and possesses pyknotic nucleus (N), mitochondria (m), and long electron-dense strands (ds). c, cilia; cm, circular muscle; lm, longitudinal muscle; pa, parenchyma. Scale bar= 1 μm. **b** The interciliary syncytium (inc) with glycogen particles (gl) and translucent vesicles (tv). sr, striated rootlets of cilia. Other abbreviations as in **a**. Scale bar = 2 μm. **c** Ciliated cell (cc) traversed by interciliary syncytium (inc) and the bulb-like structures (bs) extending above the surface of both regions and form a constriction (arrows). c, cilia; sr, striated rootlets. Scale bar = 1 μm. **d** Ciliated cell (cc) with an elongated pyknotic nucleus (N) and bulb-like structure (bs) with large vacuole (lv). Scale bar = 2 μm. **e** The basal bodies (bb) of the cilia (c) and striated rootlets (sr) lying at an angle of about 90° to the insertion level of the cilia. ds, electron-dense strands; m, mitochondria; mi, microvilli. Scale bar = 0.5 μm. **f** Interciliary syncytium (inc) covering the lateral edges of ciliated cell (cc), found beneath its basal region and covering a small part of its apical surface. c, cilia; cm, circular muscle; ds, electron-dense strands; lm, longitudinal muscle; m, mitochondria; sv, small translucent vesicle; arrow, tight junction. Scale bar = 2 μm. **g** Ciliated cell (cc) traversed by interciliary syncytium (inc) and contains mitochondria (m) and small translucent vesicles (sv). c, cilia; mi, microvilli; arrow, tight junction. Scale bar = 1 μm

**Fig. 3:**
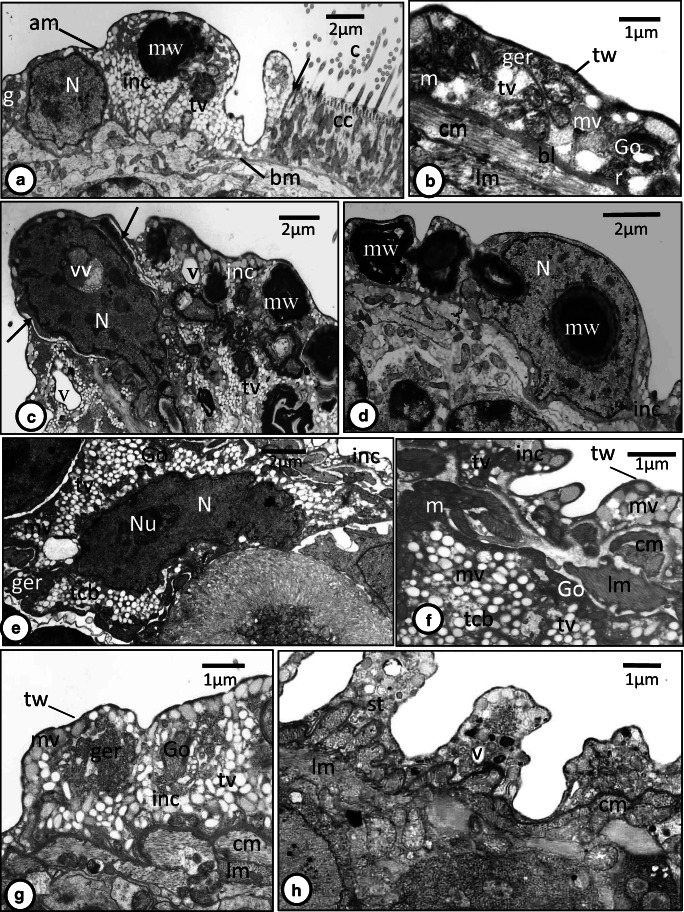
**a**‑**h** TEM micrographs of interciliary syncytium in the oncomiracidium of *Discocotyle sagittata*. **a** The interciliary syncytium (inc) is folded and its cytoplasm contains intact nucleus (N), translucent vesicles (tv), glycogen particles (gl), and multilayered whorls (mw). am, apical membrane; bm, basal membrane; c, cilium; cc, ciliated cell; tw, terminal web. Scale bar = 2 μm. **b** The smooth surface of interciliary syncytium covering the haptor. bl, basal lamina; cm, circular muscle fiber; Go, Golgi complex; ger, granular endoplasmic reticulum; lm, longitudinal muscle fiber; m, mitochondria; mv, moderately electron-dense vesicles; r, ribosomes; tv, translucent vesicles; tw, terminal web. Scale bar = 1 μm. **c** The interciliary syncytium (inc) with pyknotic nucleus (N) containing large vesiculated vacuole (vv) and surrounded by multilayered whorls (mw) and a narrow translucent layer (arrows). tv, translucent vesicles; v, large vacuole. Scale bar = 2 μm. **d** The interciliary syncytium (inc) with large granulated nucleus (N) containing a multilayered whorl (mw). Scale bar = 2 μm. **e** The tegumental cell body (tcb) with large elongated nucleus (N) containing nucleolus (Nu), granular endoplasmic reticulum (ger), Golgi body (Go), translucent vesicles (tv), and moderately electron-dense vesicles (mv). inc, interciliary syncytium. Scale bar = 2 μm. **f** The interciliary syncytium (inc) and tegumental cell body (tcb). The folded interciliary syncytium contains translucent (tv) and moderately electron-dense vesicles (mv). cm, circular muscle fiber; lm, longitudinal muscle fiber; m, mitochondria; tw, terminal web. Scale bar = 1 μm. **g** The interciliary syncytium (inc) with conspicuous granular endoplasmic reticulum, Golgi body (Go), translucent vesicles (tv), and moderately electron-dense vesicles (mv). Other abbreviations as in **f**. Scale bar = 1 μm. **h** The syncytial tegument (st) of a worm retrieved from the host 24-h postinfection with no nuclei but contains very few organelles. cm, circular muscle fiber; lm, longitudinal muscle fiber. v, vacuole. Scale bar = 1 μm

Ultrastructurally, the epidermis of recently emerged oncomiracidia of *D. sagittata* is differentiated into two regions, a ciliated cell layer and an interciliary non-ciliated, syncytial cytoplasmic layer (Figs. 1, 2b). The elongated ciliated cells are plate-like and bound laterally to the adjacent interciliary layer by tight junctions (Fig. 2b). There are numerous cilia (average length 15–18 μm), each possessing the typical “9 + 2” pattern of axonemal microtubules (Fig. 2c, d). Basal bodies of the cilia are embedded in the surface layer of the cell and each is connected at its annulus to a single relatively, long cross-striated horizontal rootlet (average 2 μm in length) lying at an angle of about 90° to the insertion level of the cilia (Fig. 2e). The basal plasma membrane and associated basal lamina form few relatively short tubular invaginations (infoldings) into the cytoplasm. The cell cytoplasm has a finely granular texture and is moderately electron dense (Fig. 2b, e). Each cell possesses an elongated nucleus (average 5.5 × 1.5 μm diameter) with an irregular outline and distinctive, highly electron-dense chromatin patches, which fill most space of the nucleus (Fig. 2). Most observed nuclei of the ciliated cells are pyknotic and the cell cytoplasm is filled with elongate mitochondria (average 3.5 μm long) that are orientated at right angles to the cell surface and a network of long electron-dense strands running parallel with the mitochondria and, in many places, contact to the cell surface between cilia (Fig. 2b‑e). Also, a few spherical translucent vesicles are seen in the cytoplasm, but no other cytoplasmic organelles are observed (Fig. 2f, g). A few short microvilli-like structures are found on the apical surface between the cilia (Fig. 2e, g). The lateral edges of the ciliated cells are interdigitated with the interciliary layer, which in many sections extends beneath the ciliated cell or sometimes extends to cover a small part of its apical surface (Figs. 1, 2f, g). Moreover, in some regions of the ciliated cells, particularly in-between the cilia and in front of the pyknotic nucleus, an apical portion of the cytoplasm extends outwardly forming a bulb-like structure containing a large vacuole filled with fine granulated cytoplasmic matrix (Figs. 1, 2c, d). In many sections, the interciliary layer, lying between the neighboring ciliated cells, extends outwardly beyond the level of the cell surface and constricts to form a nearly spherical bulb-like structure packed with secretory vesicles (Fig. 2c). In all cases, it connects with the ciliated cell via tight junctions (Fig. 3f).

The interciliary layer covering the general body surface of the oncomiracidium is nucleated and varies in thickness from 1 to 7 μm (*N* = 9) (Figs. 1, 3‑f). In most body regions, it is apparently folded (Fig. 3a, d) but becomes thinner and unfolded in the haptor region (Fig. 3b). It is bounded externally by an apical membrane and internally by a basal plasma membrane (Fig. 3b). The apical membrane is underlined by a thin, dense terminal web formed of microfilaments (Fig. 3b) while the basal plasma membrane is associated with a relatively thick and dense undulating fibrous basal lamina (Fig. 3b). Most nuclei are irregularly shaped and possess highly electron-dense, condensed chromatin patches (Fig. 3). However, some intact nuclei appear to be in the process of dissociation as they become granulated and contain large vacuoles with heterogeneous vesicles (Figs. 1, 3c), while others have a large multilayered spherical body with a central electron-dense core (Fig. 3d). Most of these nuclei are elevated slightly with the associated cytoplasm above the level of the body surface (Fig. 3c, d). Also, they are often surrounded by multilayered whorls (Fig. 3c, d) and in some cases, a narrow translucent layer is found between the nucleus and the surrounding cytoplasm (Fig. 3c). In addition to nuclei and multilayered whorls, the interciliary layer contains Golgi bodies (Fig. 3b, g), numerous mitochondria, glycogen granules, granular endoplasmic reticulum, ribosomes, and oval-shaped and circular, membrane-bound vesicles which are either translucent or moderately electron dense (Figs. 1, 3b, f, g). There are circular and longitudinal muscle layers underneath the basal lamina (Figs. 1, 3b, f, g). Nucleated cytons (tegumental cell bodies) were present underneath the muscular layers packed with translucent and moderately electro-dense vesicles similar to those in the outer interciliary layer (Figs. 1, 3e, f). The cytoplasm contains mitochondria, granular endoplasmic reticulum, and Golgi bodies that are concentrated at the periphery of the cell. However, cytoplasmic connections of these tegumental cell bodies with the outer interciliary layer could not be traced. In worms retrieved from their host 24-h post-infection, the surface layer contained very few organelles, nuclei were absent, and ciliated cells had been shed (Fig. 3h).

### Tegument of the adult

**Fig. 4:**
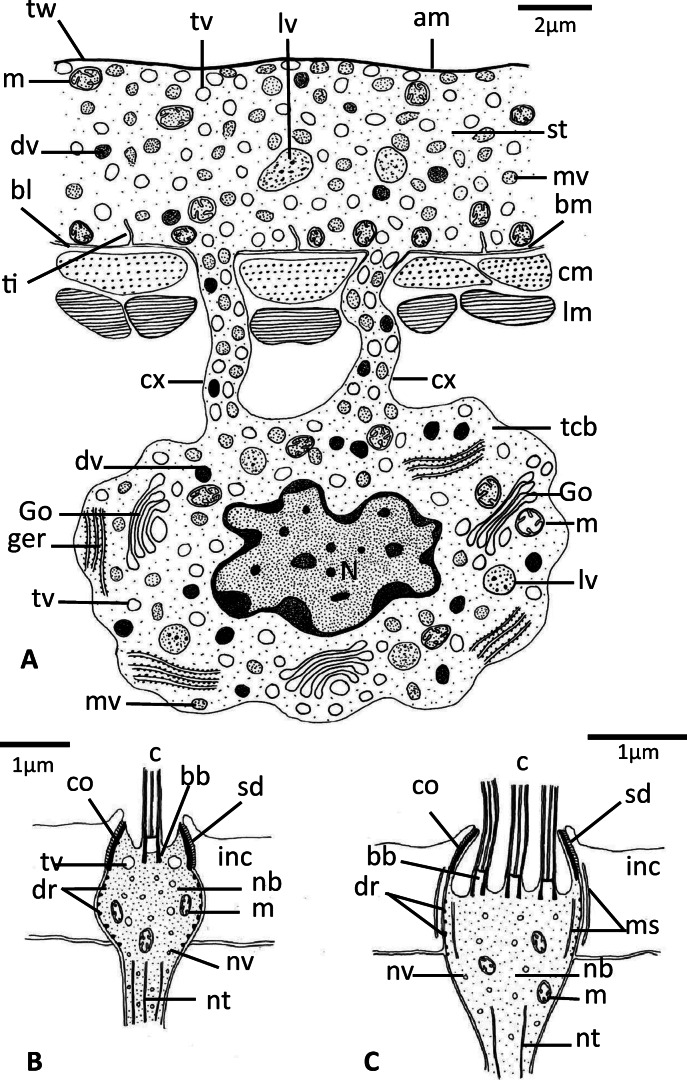
**a** Schematic drawing showing the ultrastructure of the tegument of adult *Discocotyle sagittata* in a longitudinal section. bl, basal lamina; bm, basal plasma membrane; cm, circular muscle fiber; cx, cell body connection; dv, highly electron-dense vesicle; Go, Golgi body; ger, granular endoplasmic reticulum; lm, longitudinal muscle fiber; lv, large vacuole; m, mitochondria; mv, moderately electron-dense vesicle; N, nucleus; st, syncytial tegumental layer; tcb, tegumental cell body; ti, tubular invaginations of the basal plasma membrane; tv, translucent vesicle; tw, terminal web. Scale bar= 2 μm. **b** Schematic drawing of the ultrastructure of uniciliated presumed sense organ in the oncomiracidium. bb, basal body; c, cilium; co, collar; dr, electron dense rings; inc, interciliary cytoplasm; m, mitochondrion; nb, nerve bulb; nt, neurotubules; nv, neurosecretory vesicle; sd, septate desmosomes; tv, translucent vesicle. Scale bar = 1 μm. **c** Schematic drawing of the ultrastructure of multiciliated presumed sense organ in the oncomiracidium*.* ms, membranous strands. Other abbreviations as in **b**. Scale bar = 1 μm

**Fig. 5:**
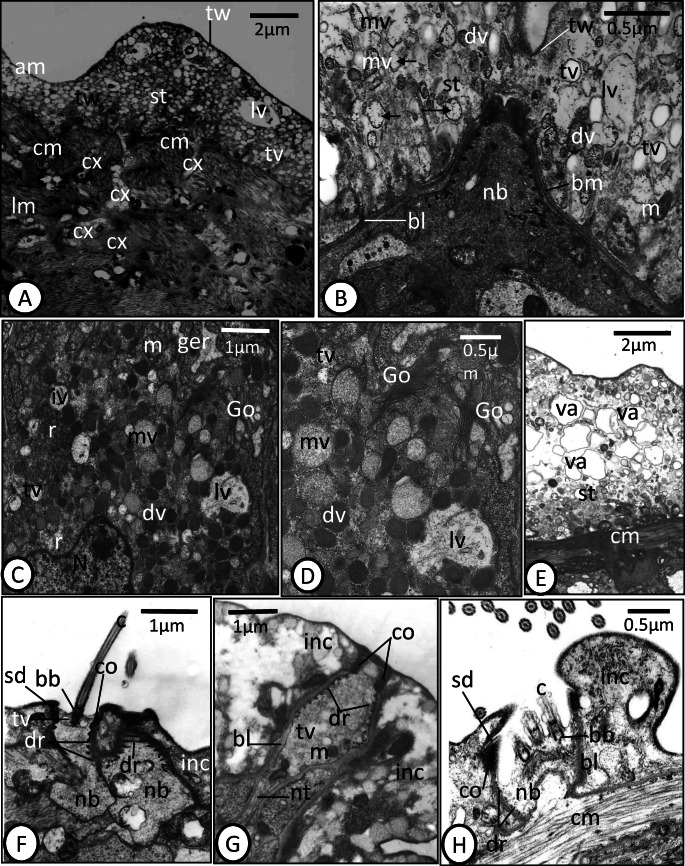
TEM micrographs of the tegument of adult *Discocotyle sagittata* (**a**‑**e**) and presumed ciliated sense organs of the oncomiracidium (**f**‑**h**). **a** The outer tegumental layer (st) with an apical plasma membrane (am), translucent vesicles (tv), and large vacuoles (lv). The subtegumental cell body connections (cx) open into the outer syncytial layer. cm, circular muscle fiber; lm, longitudinal muscle fiber; tw, terminal web. Scale bar = 2 μm. **b** The outer tegumental layer (st) with three forms of vesicles: translucent vesicles (tv), moderately electron-dense vesicles (mv), and highly electron-dense vesicles (dv). Many moderately electron-dense vesicles release their contents into the ground matrix (arrows). bl, basal lamina; bm, basal membrane; lv, large vacuole; m, mitochondrion; nb, nerve bulb; tw, terminal web. Scale bar = 0.5 μm. **c** Subtegumental cell body with irregularly shaped nucleus (N), Golgi bodies (Go), granular endoplasmic reticulum (ger), ribosomes (r), mitochondria (m), translucent vesicles (tv), moderately electron-dense vesicles (mv), highly electron-dense vesicles (dv), and large vacuoles (lv) with heterogeneous material. Scale bar = 1 μm. **d** Magnified part of subtegumental cell body cytoplasm. Golgi complex (Go) consists of flattened cisternae with associated small vesicles and large vacuoles (lv) contain heterogeneous material. Other abbreviations as in **c**. Scale bar = 0.5 μm. **e** Highly vacuolated (va) syncytial tegumental layer (st) of adult specimen maintained in vitro for 24 h. cm, circular muscle layer. Scale bar = 2 μm. **f** Uniciliated presumed sense organ. bb, basal bodies; c, cilium; co, collar; dr, electron-dense rings; inc, interciliary syncytium; nb, nerve bulb; sd, septate desmosome; tv, translucent vesicles. Scale bar = 1 μm. **g** Nerve bulb of uniciliated sense organ surrounded by basal lamina (bl) and contains collar (co), electron-dense rings (dr), mitochondria (m), translucent vesicles (tv) and neurotubules (nt), inc, interciliary syncytium. Scale bar = 1 μm. **h** Multiciliated presumed sense organ with 3 cilia (c) and their basal bodies (bb). The nerve bulb (nb) connects with interciliary syncytium (inc) by septate desmosomes (sd) and has a collar (co) and electron-dense rings (dr). cm, circular muscle fiber. bl, basal lamina. Scale bar = 0.5 μm

The general body tegument of subadult and adult *D. sagittata* is composed of an external syncytial layer, connected to subtegumental cell bodies (cytons) through cytoplasmic connections traversing the tegumental muscle layers (Figs. 4a, 5a). The syncytial tegumental layer varies in thickness from 0.5 to 7.5 μm. The apical plasma membrane is lined internally by a dense terminal web of microfilaments similar to that in the oncomiracidium (Figs. 4a, 5a, b). The basal plasma membrane is underlined by a conspicuous, moderately electron-dense basal lamina that form numerous relatively short tubular invaginations into the outer syncytial tegumental layer (Figs. 4a, 5a, b). The outer syncytial layer has no cytoplasmic organelles except mitochondria that are concentrated close to the basal plasma membrane (Fig. 5b). Spherical membrane-bound vesicles fill much of the syncytial layer, but they vary in appearance. Most vesicles are translucent, but some are moderately electron dense while a few are highly electron dense (Figs. 4a, 5a, b). Some large vacuoles are visible, while some of the moderately electron-dense vesicles were captured releasing their electron-dense particles into the ground substance (Fig. 5b). The tegument musculature consists of several layers of circular and longitudinal muscle fibers (Fig. 5b). Each subtegumental cell body possesses a well-developed nucleus and its cytoplasm is filled with Golgi bodies, ribosomes, granular endoplasmic reticulum, mitochondria, and characteristic secretory vesicles with different forms as well as irregularly shaped vacuoles with heterogeneous contents (Figs. 4a, 5c). Each Golgi complex consists of 3–8 flattened cisternae with associated small vesicles (Fig. 5c, d). Cytoplasmic connections carrying secretory vesicles extend from these cells where they open into the outer syncytial layer (Figs. 4a, 5a). There were no detectable differences in the tegument of those specimens that had been naturally dislodged from their host and those recovered in screening water, although there was an increase in surface layer vacuolation of control specimens that had been maintained in vitro for 24 h (Fig. 5e).

### Presumed surface sense organs of the oncomiracidium and adult

Two types of presumed sensory structures were detected on the body surface, particularly common around the anterior region of the oncomiracidium. These are a uniciliated receptor and compound multiciliated receptor, both of which penetrate the interciliary syncytial layer (Figs. 4b, c, 5f, g, h). Only the uniciliated receptor was observed on the body surface of the anterior region of the adult. However, in some sections, two separate but closely attached uniciliated receptors could be seen in the interciliary syncytial layer of the oncomiracidium (Fig. 5f). The uniciliated receptor consists of a nerve bulb anchored to the tegumental layer by annular septate desmosomes and bears a single (9 + 2) cilium measuring 2.7 μm (dia.), with a normal basal body but without rootlet (Figs. 4b, 5f). At the level of the basal body, a layer of electron-dense thickening (a collar) is found on the inner surface of the bulb (Figs. 4b, 5f, g) while at the level posterior to the basal body, the bulb is supported by 8–10 annular, electron-dense rings (Fig. 5g). The bulb contains a homogeneous, moderately electron-dense matrix in which are embedded mitochondria, neurotubules, and translucent vesicles (Figs. 4b, 5f, g). In some sections, the nerve bulb of the uniciliated receptor was seen in continuity with a neuron containing a relatively large spherical nucleus. The multiciliated receptor appears to consist of a single nerve bulb terminating with at least three cilia with the typical structure (9 + 2) and connects to the neighboring syncytial layer by septate desmosomes (Figs. 4c, 5h). A collar, basal bodies and thin, electron-dense rings are present, but rootlets were not seen (Fig. 4c). Also, the nerve bulb contains membranous strands close to the dense rings (Fig. 4h). Similar membranous strands are seen outside the bulb in the syncytial tegumental layer (Fig. 4c).

## Discussion

Typical of monogeneans, the tegument of adult *Discocotyle sagittata* is composed of a surface syncytial cytoplasmic layer separated from underlying tegumental cell bodies by a basal lamina complex and muscle blocks. The present findings, together with previous studies, suggest a distinct variation in the epidermis ultrastructure among oncomiracidia of different monogenean species. The oncomiracidial epidermis of *D. sagittata* is differentiated into two regions, a ciliated cell layer and an interciliary, syncytial cytoplasm, both of which are nucleated. Moreover, the interciliary syncytium extends between the ciliated cells, covering their lateral sides, and sometimes part of their basal and apical surfaces. Subepidermal cell bodies containing secretory vesicles similar to those in the interciliary syncytial layer were seen among parenchymal cells with their cytoplasmic processes extending upwards, in close contact with the subtegumental muscles. Previous studies have shown that the two-layered tegumental structure of monogeneans develops from a primitive epithelium in which a nucleated apical layer becomes connected via cytoplasmic processes to underlying subtegumental cells (Lyons 1973; Smyth and Halton 1983; Bereiter-Hahn et al. 1984; Cable and Tinsley 1992). Three categories can be recognized for this switch in tegument morphology in the oncomiracidia of monogeneans so far studied. In the first, nuclei of both ciliated cells and interciliary cytoplasm are shed from the surface layer and the epidermis becomes a syncytial tissue during the later stages of embryogenesis as occurs in *Entobdella solea* and *Pseudodiplorchis americanus*(see Lyons 1973; Cable and Tinsley 1992 respectively). In the second category, nuclei of the ciliated cells and interciliary syncytium of the oncomiracidium remain distinct and the switch in tegument morphology occurs after the oncomiracidia hatch as in *D. sagittata* (present study), *Polystoma integerrimum* and *Polystoma pelobatis*(see Fournier 1979), and *Zeuxapta seriola*(see Rohde 1998). The third category is represented by oncomiracidia of *Euzetrema knoeffleri*, where the nuclei remain distinct in the ciliated epidermis but those of the interciliary epidermis are lost during embryonic development (Fournier 1976). The monogenean *Polystoma* spp. are anuran parasites characterized by a dimorphic life cycle; the normal slow-growing form migrates to the host’s bladder when the tadpole metamorphoses, whereas the fast-growing neotenic form matures on the gills of the tadpole; only the slow-growing form exhibits the delay in tegumental transformation (Fournier 1979). There is no obvious ecological or behavioral explanation to interpret this delayed switch in the life cycle of the mazocraidids *D. sagittata* and *Z. seriola* but perhaps this phenomenon is common and is just a reflection of the rarity of ultrastructural detection of surface nuclei. Persistence of nuclei of the ciliated cells and interciliary syncytium of the oncomiracidium of *D. sagittata* and other monogeneans indicates that these cytoplasmic layers continue to perform their synthetic and physiological activities which are controlled by these nuclei. The nuclei of ciliated cells may have a role in controlling shedding of the ciliated cells as soon as the oncomiracidium makes contact with the gills.

A characteristic feature of the interciliary layer of the oncomiracidium of *D. sagittata* is the presence of numerous, large electron-dense whorls. Moreover, some intact nuclei contain membranous whorls or large vesiculated vacuoles. Most of these apparently dissociating nuclei project slightly above the surface of the parasite with a narrow layer of cytoplasm. Also, the nuclei of the ciliated cells appear pyknotic. All these features tend to be indicative of membrane turnover suggesting that the switch in epidermis morphology is already initiated at this stage. This transition appears to be complete in worms retrieved from their host 24-h post-infection where the surface syncytial tegumental layer contained very few organelles and had no nuclei, and their ciliated cells were shed. In *P. americanus*, asynchronous shedding of ciliated cells occurs 1–2-h post-infection and exciliation involves coalescence of basal vacuoles to form a large cavity which enlarges under the ciliated cell causing rupture of the lateral septate desmosomes (Cable and Tinsley 1992). In the present study, none of these vacuoles was observed suggesting that the shedding mechanism in the oncomiracidium of *D. sagittata* could be different. In the oncomiracidium of *E. soleae*, Lyons (1973) found no vacuoles between the ciliated cells and the underlying layer of discontinuous “presumptive adult tegument” and suggested that the process of shedding is undertaken under nervous control since the ciliated cells are shed when the oncomiracidium attaches to the fish host. The author presumed that either the ciliated epidermis or the presumptive adult layer secretes a substance that acts on the intercellular cement. A similar shedding mechanism could occur in the oncomiracidium of *D. sagittata* since the interciliary layer extends beneath the ciliary cells and covers their lateral surfaces. The large subtegumental cell bodies found underneath the subtegumental muscle bands resemble those of other monogeneans where their cytoplasmic processes fuse the interciliary cytoplasm that replaces the shed ciliated cells. The presence of cytoplasmic bulb-like structures of both the ciliated cells and interciliary cytoplasm of the oncomiracidium of *D. sagittata* are good evidence of pinching off some parts of the epidermal cytoplasm and subsequently support the hypothesis that the switch in epidermis morphology occurs at this stage. In the digenean miracidium of *Fasciola hepatica*, large vacuoles are formed between the base of the cilia and the underlying subepidermal layers, followed by expansion of the cytoplasmic interciliary ridges to replace the lost ciliated cells (Southgate 1970).

The cilia of the oncomiracidia of *D. sagittata* (present study), *E. soleae*(see Lyons 1973), *Euzetrema knoeffleri*(see Fournier 1976), *P. integerrimum*(see Fournier 1979), and *Pseudodiplorchis americanus*(see Cable and Tinsley 1992) all have only a single horizontal cross-striated rootlet, while the cilia on the oncomiracidia of the monocotylids, *Neoheterocotyle rhinobatidis* and *Monocotyle spiremae* have two rootlets, a well-developed horizontal rootlet and a much less-developed vertical rootlet (Rohde et al. 1998). However, contrary to the vertical rootlets of turbellarians, which originate from the basal bodies, the vertical rootlets of these monocotylids originate from the basal parts of the horizontal rootlets (Rohde et al. 1998). It was considered unlikely that they were homologous in the two groups and were therefore termed “false vertical rootlets” in the monocotylid species (Rohde et al. 1998). In the oncomiracidium of the polyopisthocotylean *Z. seriolae*, vertical rootlets are missing, although bundles of fine filaments extend from the basal bodies into the cytoplasm of the epidermal cells, straddling horizontal rootlets of cilia in the same longitudinal rows (Rohde 1998). These filaments may anchor the cilia in the cell body. In the ciliated cells of *D. sagittata*, long electron-dense strands were observed running parallel with the mitochondria and, in many places, contact the cell surface between cilia; similar structures were described in the ciliated cells of *E. soleae*(see Lyons 1973) and *P. americanus*(see Cable and Tinsley 1992). These strands may serve as a supportive skeleton that prevent ciliated cell cytoplasm from collapse during locomotive strikes of the cilia. Secretory products derived from these strands may protect the apical part of the cell since it lacks a terminal web.

The body tegument of subadult and adult *D. sagittata* follows the general pattern described in other monogeneans (Smyth and Halton 1983; El-Naggar et al. 1991; Cribb et al. 2003; Hodová et al. 2010; Poddubnaya et al. 2016). There were no detectable differences in the tegument of adult and subadult specimens (with a single pair of clamps on the opisthaptor) of *D. sagittata* which had been naturally dislodged from their host and recovered in screening water, although there is an increase in the surface layer vacuolation of adult specimens which had been maintained in vitro for 24 h. The present findings indicate that the physiological activities of the tegument may continue for a specific period after natural dislodgement of the worms and during this period, they might be able to reattach to a host.

The syncytial tegumental layer of adult *D. sagittata* contains membrane-bound vesicles, most of which are translucent. These vesicles are likely manufactured by Golgi complexes in association with the granular endoplasmic reticulum. There was evidence that some vesicles release their contents into the ground substance of the syncytial layer. Possibly this contributes to the formation of the fibrous terminal web, which may have protective and supportive functions. Exocytosis of the contents of the tegument vesicles was reported in many other monogeneans, for example, *Allodiscocotyle diacanthi*(see Ramasamy et al. 1995) and it has been suggested that they are involved in glycocalyx maintenance or the provision of a protective layer of mucus over the apical plasma membrane to minimize mechanical osmotic and immunological damage to the tegument surface.

Uniciliated and multiciliated, presumed sensory receptors were detected penetrating the interciliary syncytium of the oncomiracidium while only the uniciliated receptor was observed on the body tegument of adult *D. sagittata*. Similar sensory receptors have been recorded in other monogeneans. Uniciliated receptors were reported in the adult and oncomiracidium of *E. solea*, adult *Gyrodactylus* spp., adult *Leptocotyle minor*, juvenile *Amphibdella*, and adult *Diclidophora* and *Polystomoides* spp. (see Smyth and Halton 1983). Most of these receptors are located at the anterior region of both the adult and oncomiracidia, possibly serving as tangoreceptors or rheoreceptors. The compound multiciliate receptors observed in the head region of adult and larva of *E. solea*, spike sensilla of *Gyrodactylus* spp., and adult *Polystomoides* may have chemosensory or tangoreceptive functions (Smyth and Halton 1983). There may be more types of undetected sensory structures in the oncomiracidium and adult of *D. sagittata*; this requires silver nitrate staining histology and further electron microscopy.
